# The ethics of artificial intelligence use in university libraries in Zimbabwe

**DOI:** 10.3389/frma.2024.1522423

**Published:** 2025-01-17

**Authors:** Stephen Tsekea, Edward Mandoga

**Affiliations:** ^1^Department of Information Science and Records Management, Zimbabwe Open University, Harare, Zimbabwe; ^2^Department of Teacher Development, Zimbabwe Open University, Harare, Zimbabwe

**Keywords:** artificial intelligence, higher education, ethics, university libraries, higher education integrity

## Abstract

**Introduction:**

The emergence of artificial intelligence (AI) has revolutionised higher education teaching and learning. AI has the power to analyse large amounts of data and make intelligent predictions thus changing the whole teaching and learning processes. However, such a rise has led to institutions questioning the morality of these applications. The changes have left librarians and educators worried about the major ethical questions surrounding privacy, equality of information, protection of intellectual property, cheating, misinformation and job security. Libraries have always been concerned about ethics and many go out of their way to make sure communities are educated about the ethical question. However, the emergence of artificial intelligence has caught them unaware.

**Methods:**

This research investigates the preparedness of higher education librarians to support the ethical use of information within the higher and tertiary education fraternity. A qualitative approach was used for this study. Interviews were done with thirty purposively selected librarians and academics from universities in Zimbabwe.

**Results:**

Findings indicated that many university libraries in Zimbabwe are still at the adoption stage of artificial intelligence. It was also found that institutions and libraries are not yet prepared for AI use and are still crafting policies on the use of AI.

**Discussion:**

Libraries seem prepared to adopt AI. They are also prepared to offer training on how to protect intellectual property but have serious challenges in issues of transparency, data security, plagiarism detection and concerns about job losses. However, with no major ethical policies having been crafted on AI use, it becomes challenging for libraries to full adopt its usage.

## Introduction

A tremendous shift is changing teaching and learning in higher education due to the coming of artificial intelligence (AI) technologies (Hlongwane et al., 2024). AI is a group of technologies that allow computers to solve problems dynamically (Harisanty et al., 2023). Tredinnick (2017) defined AI as a cluster of technologies and approaches to computing focused on the ability of computers to make flexible rational decisions in response to unpredictable environmental conditions. Artificial intelligence can be used to improve the quality of service delivery in many organizations. Schlagwein and Willcocks (2023) noted that AI seeks to make computers do what human minds can. AI involves data analytics, machine learning, big data, and natural language processing. Despite the powerful capacity of AI to help the academic community write assignments, projects, and other technical assistance, there is a notable gap in understanding the ethical dimensions such as allowing students to cheat through plagiarism, disinformation, transparency, accountability, fairness, and discrimination. Students use AI technologies such as ChatGPT to write assignments while staff can use research applications tools like Research Rabbit, Jenni AI, Connected Papers, and Grammarly while failing to acknowledge the use of such. Using such AI technologies has led to more questions than answers on their possible ethical aspects. Bélisle-Pipon et al. (2021) concurred that advances in AI have come with concerns about ethical, moral, legal, and social issues.

## Background

Nalini (2019) defined ethics as moral principles governing the behavior or action of an individual or group. Ethics help people, families, society, professions, and organizations to have a set of principles or beliefs determining what is right or wrong. The issue of ethics has attracted research from various researchers in different disciplines such as education, commerce, social science and arts, and culture. Ethics encompasses moral philosophy. These include the values that determine behavior or how human beings ought to behave. Every action or experience we have in life is guided by ethics (Prasad, 2019). Ethics have their roots in philosophy and study the difference between right and wrong. Theoretical and applied ethics are the main two forms of ethics. There are two bodies of ethics, that is, normative and applied ethics. Normative ethics focuses on studying actions that make right or wrong while applied ethics looks at ethical issues of private and public life (Prasad, 2019).

The need to study ethics came in the early 2000s when researchers and other higher education stakeholders began to worry about the ethical issues posed by AI systems (Tawfeeq et al., 2023). This was due to the growth in the use of AI in teaching and learning. Assessment became challenging due to academic misconduct such as plagiarism and buying essays or assignments. According to Rudolph et al. (2023), AI applications such as ChatGPT have the potential to produce outstanding text within a matter of seconds leading to much buzz and doomsday predictions regarding student assessment in higher education. The academic community saw massive cheating and plagiarism being aided by artificial intelligence. Other latest applications such as Meta which comes through WhatsApp are also being used by students to do their research. The use of AI in educational settings may cause students to become less adept at critical thinking since they may come to rely excessively on the responses that are generated by AI rather than coming up with their own ideas (Mhlanga, 2023). Unethical behavior such as the spreading of misinformation and disinformation has also been witnessed leading the academic community to question the ethical use of AI. The ethics question centers on the moral obligation or duties of an AI application. Siau and Wang (2020) noted that the ethics of AI is part of the ethics of advanced technology that focuses on robots and other artificial intelligence agents. Ethics of AI looks at the moral obligation and duties of AI applications and their creators.

The study of the ethical implications of AI use in Libraries is quite pertinent. This is so because these AI tools and applications are increasingly prevalent in Libraries and their use has the potential to transform the way libraries operate (Mishra, 2023). Some of the processes that Libraries are using technologies include resource processing, client relationship management, and teaching (Hodonu-Wusu, 2024). AI algorithms can also be used to monitor library reader's reading preferences. Such a personalized approach saves users' time and helps libraries collect data on users' preferences thus providing the right resources. Chatbots are also being used to communicate with clients. Chatbots are computer programmes that can simulate an intelligent conversation, through text, speech, or potentially through an embodied representation (Cox et al., 2018). Embracing such technologies comes with significant ethical issues. While in the developed world there are great strides in introducing ethical policies and standards for the usage of AI, developing countries seem to be lagging.

In Zimbabwe ICT regulation is through the National ICT Policy for Zimbabwe which was launched in March 2024. At the time of writing this research, the country had no specific AI policy. AI adoption is still in its infancy even though there are a lot of benefits that accrue from its use (Hlongwane et al., 2024). Only a few countries in Africa like Kenya, South Africa, Ethiopia, Nigeria, Ghana have laid down a solid foundation for the integration of AI into their education systems (Hlongwane et al., 2024). AI is being used in higher education to create and supplement content, for example at the Zimbabwe Open University, which specializes in distance education, AI applications like Teaching Assistant Pro are used. Libraries have the potential to benefit from AI through enhanced discoverability and accessibility to their resources and services. In higher education libraries in Zimbabwe, AI applications that have been adopted include chatbots to improve communication between librarians and patrons. AI is also used to detect plagiarism with such tools as Turnitin being popular. The challenging ethical issue surrounding the use of these AI applications is personal data privacy. However, very few African countries have enacted legislation to protect data (Afolabi, 2024).

## Purpose of the study

This research looks at the ethics of the use of artificial intelligence in libraries. The paper delineates how librarians are positioning themselves for the AI world.

### Problem statement

The main goal of university libraries is to support teaching and learning in universities. They are expected to offer advanced services that support student learning experiences. Libraries support students and staff with such services as literature searching, referencing, and anti-plagiarism. They also offer resources and services to all students regardless of mode of learning. However, libraries found themselves serving a clientele base that is adopting AI at great length. Libraries also do personalized and targeted marketing services where they use algorithms to study their readers' preferences and behavior. Such access to huge amounts of data, intellectual property resources and a clientele base made of students who may want to cheat their way to success, results in a need for libraries to worry about the ethical issues surrounding the use of AI. This is so because there are many ethical implications of AI such as surveillance, transparency, and confidentiality. It is therefore necessary to interrogate the potential extent of disruption that these technologies can offer. Parry (2012) noted that though there has been such a surge in technologies, it is necessary to investigate the state of preparedness of higher education in addressing ethical issues. If such interrogation is left unattended, there is a possibility of continued adoption of these technologies while distorting higher education's moral aspects. Such ethical considerations may also ensure equity and inclusivity in the use of technology in higher education.

### Study objectives

- To analyze the AI applications used in libraries;
- To determine the level of adoption of AI in libraries;
- To analyze the policies and practices surrounding the use of AI in higher education; and
- To determine librarians' perceived ethical and moral issues with AI.

### Literature review

There are various theories of ethics and how ethics can be classified. Notable among these is one classification put forward by Schlagwein and Willcocks (2023) who noted that ethical theories can be classified into Deontological theories, which focus on the actions, and Teleological ethics which focus on the results achieved by actions. Deontological theories are referred to as rule-based. Baron et al. (1997) noted that deontological ethics prioritize the process over the outcome in moral considerations. Teleological ethics, on the other hand, focus more on outcomes. Schlagwein and Willcocks (2023, p. 233) noted that “a fair process is nice to have, but ultimately, what ethically matters are improvements in the relevant outcomes.” The question that scholars of artificial intelligence are still grappling with is whether AI is to be judged on deontological or teleological ethics. There seems therefore to be a gap in the state of preparedness of higher education in addressing ethical concerns despite their adoption.

In libraries, just like any other field, AI is just coming into focus. The use of AI is still in its infancy in most libraries, particularly in developing countries. Cox et al. (2018) researching the likely impact of AI in libraries, noted that one of the areas that libraries are likely to benefit from is communication with their patrons through the use of chatbots. These chatbots can also be called digital assistants, virtual agents or intelligent agents. There are mainly two types of chatbots and the categories can vary for example, there can be guided chatbots and Frequently Asked Questions (FAQ) chatbots. According to Mckie and Narayan (2019), a guided chatbot responds to the context of the conversations and asks the user questions. A FAQ chatbot responds without understanding the context of the conversation and does not retain previous conversations with the user (Mckie and Narayan, 2019). Baez et al. (2021) categorized chatbots into task-oriented, which are designed to provide services related to specific content in a domain and chit-chat bots, designed to engage in conversations.

It is noted in the literature that one fundamental ethical concern of using AI-generated work is the issue of bias and prejudice in the data used to train the algorithms. This has worried the scientific community as biased data can lead to biased conclusions. Campolo et al. (2017) raised the issue of the biases of AI by popping the question: can AI be trusted to make fair decisions? Chat Generative Pre-trained Transformer (ChatGPT) is one AI application that has gained a tremendous rise since its launch in November 2022 (Dwivedi et al., 2023). It responds to user requests or queries. ChatGPT relies on large datasets to train its algorithms and these data sets may contain biases. In academia, Liebrenz et al. (2023) noted that ChatGPT is used by students to formulate university essays, and scholarly articles with reference, to compose music, to write poetry and other functions. The question that scholars are worried about is the ethics of using generative AI in conducting research or writing a journal paper or book chapter.

Privacy and data security are also key considerations that are noted in literature which the scientific world needs to consider when using generative AI. Cox et al. (2018) claimed that manipulation and privacy are serious ethical issues that the scientific world needs to interrogate. Thabit and Jasim (2017) cautioned that privacy, security, responsibility, and the influence of human social skills are some of the ethical concerns raised by AI applications such as ChatGPT. In the library and information science profession, researchers have to start questioning the privacy and security implications of using generative AI (Soni, 2024). Since libraries handle large sums of data generated through interacting with patrons on social media platforms and in chatbots, the privacy of such data becomes of serious concern to the scientific community.

Dowling and Lucey (2023) noted that using AI in research makes it difficult to get informed concerns. Similarly, Rapp et al. (2023) noted that these AI technologies mislead users into believing that they are communicating with a human thus raising the important issue of informed consent. Informed consent is one of the critical ethical issues in research that promotes transparency. Cox et al. (2018) raised the challenge of cost as AI is likely to be expensive for many industries. The digital divide will likely continue between the North and the South. Poor developing communities will continue to lag in terms of adoption and use of AI due to cost. Mhlanga (2023) also concurred that with the coming of AI, there is a possibility of further division between the rich and poor nations in the adoption of AI.

In a study on the ethical implications of ChatGPT, Tawfeeq et al. (2023) found that many organizations still have not developed policies and standards on the use of AI. They recommended constant review and modification of such regulations to promote the ethical use of ChatGPT. Some study, however, reveals that ChatGPT models have the potential to perpetuate racial, gender and religious prejudices in their replies (Mhlanga, 2023).

### Methodology

The research paradigm followed in this study is interpretivist and a qualitative approach was taken. The approach was chosen because of its capability to allow analysis of words and their contextual meanings. Researchers such as Hlongwane et al. (2024) also used the qualitative approach to explore the phenomenon of ethics of AI use because of its capability to facilitate an in-depth comprehension of complex phenomena like AI integration in higher education teaching and learning. Researching ethical dilemmas faced by students in using AI, Mutanga et al. (2024) also took an interpretivist approach where they used a thematic approach to analyze qualitative data. Qualitative research as noted by Addo and Eboh (2014) offers rich, detailed and context-specific data that go beyond surface-level descriptions. This gives the researchers room for getting rich data through a deeper understanding of the participants' perspectives, meanings and lived experiences. Semi-structured interviews and document analysis were used to collect data. Semi-structured interviews offer an opportunity for a researcher to get the context for informant behaviors. One can also be able to get real-life scenarios being expressed through emotions.

Thirty (30) participants who were purposively sampled for the study were drawn from librarians and academics in the higher education sector in Zimbabwe. Twenty four (24) librarians and six (6) lecturers were successfully interviewed. These were drawn from three selected universities in Zimbabwe namely Great Zimbabwe University, Bindura University of Science Education and the Zimbabwe Open University. There were an equal number of participants at each institution (six librarians and two lecturers). Interviews were conducted between May and July 2024. Informed consent, where the participants were requested for their consent, was granted before the interview. The researchers used audio recordings and written notes during the interviews which were carried out in participants' offices and some via online platforms. An interview guide was used by the researchers and the interviews lasted approximately 20–30 min. Participants used English and Shona depending on their preference.

## Findings

While there has been a lot of debate surrounding the use of AI, participants noted that their institutions were yet to fully adopt these applications. The rate of adoption was reported to be very in Zimbabwe due to cost challenges. Participants from the three selected universities all pointed out a slow uptake of AI in higher education in Zimbabwe. There were also challenges with the right personnel, cost of infrastructure, fear of job losses, and attitude problems. This showed that all the institutions that participated in the study were ill-prepared to adopt AI. The data showed that the most commonly used AI application in Zimbabwean libraries was the Chatbots and virtual assistants and anti-plagiarism applications. Chatbots applications are used in libraries to manage routine queries from clients. Anti-plagiarism applications are used to detect plagiarism of assignments and other research work. However, it was shown that only one university had managed to subscribe to Turnitin's AI detection module. The participants also indicated that they have not employed any advanced AI data analysis applications to monitor readers' behavior but were willing and ready to adopt such. One participant said “*We would like to use AI in such processes as cataloging and classification, information literacy training and stock taking but we have not yet adopted any AI system.”* Librarians who were at the senior levels showed some appreciation of what AI is as they are involved in information literacy skills training and general administration of the libraries.

When asked about the adoption of AI in their institutions, all the participants agreed that they were yet to fully adopt the use of AI in their libraries. Some of their responses were as follows:

*To me, AI is just a buzzword, we have not started implementing any AI applications in our library*.

*We hear about it in literature. We have been trained but have not fully adopted it*.

*We have started to use it (AI), particularly in anti-plagiarism check and chat services*.

All the participants agreed that the adoption of AI was a noble idea for university libraries. However, some lamented the challenge of effective capacity building of librarians so that they could effectively use some of the AI. One of the senior librarians noted the challenge of high implementation costs, “*We would like to adopt AI just like other libraries in developed countries but our authorities always talk of budgetary challenges. So even including such in our annual plans is a dream*.”

Librarians noted that some of the intelligent applications that are being used include ChatGPT, Alexa, and Tawk2. These are used as chatbots for communication purposes to assist users in real-time and to assist the user looking for information. Higher education institutions handle a lot of data coming from communicating with clients. Protection of such data becomes therefore a major ethical priority. Participants noted that despite AI assisting them in generating massive data, they still respect their patrons by maintaining privacy through not releasing their identity. Libraries users of such intelligent platforms such as ChatGPT have to know the surety and privacy issues of the data that is gathered through their interaction. One participant said:

*We generate a lot of data through interacting with clients. I believe it is our responsibility not to divulge or share such information. I believe the maintenance of privacy is key*.

Another participant said:

*Students provide us with a lot of data. Some of which can be personal. There is definitely a need to keep such information safe. But the challenge that is there is that we don't have any policies on the use of these platforms*.

One of the major issues noted by participants was the accuracy of the information generated by AI. In a world where disinformation and misinformation is rampant on the Internet, relying on AI to write an assignment presents serious ethical challenge. One participant noted that:

*Students believe everything that is generated on the Internet is factual. Some are not well educated in the concept of garbage in garbage out. Whatever they get on these AI tools they just accept*.

Participants were also asked about policies and standard operating procedures in their institutions. We noted that many institutions were still to have fully functional policies and procedures. However, they concurred that such policies were very crucial in maintaining the integrity of higher education. Participants were worried about the misuse of AI in cyber attacks, disinformation and misinformation, political attacks and phishing. One systems librarian reported that:

*I often receive students who visit me to enquire about their accounts on such platforms as social media and e-learning platforms being hacked. Students report that they sometimes receive unsolicited content on their accounts*.

Participants noted that information generated through AI needs to be treated with caution. One librarian at one of the universities in Zimbabwe noted that:

*We see students doing their assignments using these AI applications. Some of their favorite applications here are POE and ChatGPT. We wonder whether they take time to do some evaluation of the information that they would have taken from the net (Internet)*.

In addition, participants also highlighted the challenge of inclusivity and bias. One participant from a university specializing in distance education noted that:

*When it comes to inclusivity, I think these AI applications are for those who can afford data and technology literate. We have students in some remote areas that may want to try and access our library resources and services such as the anti-plagiarism software, Turnitin but will sometimes fail due to internet connectivity and the cost of data*.

This was also similar to the views expressed by one academic who said, “*The assignments that we are getting are somehow pointing to students using AI. However, its use varies. There seem to be some students who are conversant in it [AI] while some are yet to. There is therefore a risk on discrimination.”*

## Discussion

This research has shown that librarians are worried about the challenge of evaluation of information taken from the internet. This is similar to what was noted in literature by such scholars as Mhlanga (2023) who observed that academics need to be cautious about materials or information generated through AI since there is no guarantee that such information will be accurate, trustworthy, and credible. It is therefore necessary for lecturers and other researchers to always check the accuracy of the materials submitted by students since there is a huge possibility that due to gullibility, the likelihood of duplication of information found on such platforms as ChatGPT and of late Meta is correct. There is a need for massive awareness campaigns to educate students on the need to be critical of the content they get from any AI-generated applications.

This research has shown that AI may create a digital divide where those who can afford the technologies will continue growing at a faster rate than the poor ones. Such gaps create inequality in access to technologies. This is similar to the findings of Hodonu-Wusu (2024) who advocated for libraries to actively engage in the use of ethics in AI so as to promote equal access, unbiased and transparent information flow. The digital divide has shown that most institutions in Zimbabwe are ill-prepared for the adoption of AI since there is no favorable infrastructure to support its adoption and effective use.

Another key ethical issue that this research has raised is the publishing of research by scholars. Participants from this study have shown that the use of AI in publishing creates challenges. This is however contrary to the views of Schlagwein and Willcocks (2023) who pointed out that in publishing it is permissible to use AI as there is no need to police authors. They further noted that it is the responsibility of the author to be ethical but in the principle of transparency, authors should declare their use of AI. Ethical behavior is part of scholarship and authors are obliged to reflect on and explain their methods and ethics applied in their work (Schlagwein and Willcocks, 2023). In the history of librarianship, the guiding principles have always been anchored on the promotion of intellectual freedom, privacy and equity so scholars can be allowed to use AI. However, with the coming of these massive technologies, there is a need for a paradigm shift in the creation of policies and procedures on the ethical issues surrounding the use of AI. Huang et al. (2021) cautioned that given that technological transformations are happening beyond the everyday responsibilities of librarians as a part of a larger economic shift, then the question of ethics needs to move beyond mere professional statements to include practices grounded in strong ideological practices.

Libraries have always had an ethical and equitable promise to their users and they have to worry about AI ethics (Hodonu-Wusu, 2024). Though this study has noted that AI is still in its infancy in most higher and tertiary education institutions in Zimbabwe, interrogating the ethical dimensions of these must be discussed now as not doing so has a potential of harming people. Paying attention to the ethics and morals of AI is very important in that it enables the effective adoption of AI in education. Libraries have always had an ethical and equitable promise to their users and they have to worry about AI ethics. Popenici and Kerr (2017) argue that an academic perspective is needed to question the quick tendency to turn to technology for answers, especially without being ready. They worry about overreliance on technology neglecting the richness of human knowledge and perspective. Students, lecturers, and other researchers in the higher education sector need to adopt the latest technological movements such as AI. However, there is a need for massive awareness campaigns of the dangers of unethical use of AI which has the potential to wipe out the integrity of higher education. Further development and use of AI can only be enhanced if ethical issues are paid attention to and proper policies and guidelines are enacted.

This study recommends training higher education practitioners in using AI in teaching and learning to be fully prepared for complete adoption. There is also a need for the development of policies and procedures that govern the use of AI. Taddeo et al. (2019) also noted the need for proper regulation, development and improved cybersecurity measures. Campaigns for the negative consequences of AI use should be started at the orientation and induction stages of students and staff respectively to create awareness of the dangers of AI.

This study further recommends the need for maintaining privacy on user-generated data. While AI has the potential to understand more about user preferences and reading behavior, there is a need for proper management of data. There is a potential of the generation of large amounts of data, including personal information. Libraries need to have proper ways of being transparent about data that is collected and how they are going to store it. Libraries can also come up with AI policies that enforce the use of consent forms or agreements with their users. Another recommendation is on the urgent need for crafting best practices and policies that will allow AI of culturally sensitive systems.

## Conclusion

Artificial intelligence presents an opportunity for solving some of the world's problems. In higher education, AI use is rising tremendously, either by students or staff through AI algorithms and data analytics applications. This study has shown that AI is being used for student, instructional, and institutional support. This study has shown that AI has the power to revolutionize Library operations. AI can allow access to vast amounts of information and help in the improvement of personalized learning thereby facilitating the acquisition and retention of knowledge by students and staff. However, with its rise, the morality of it is being questioned. The ethical issues surrounding the use of AI have become a critical concern in higher education. This qualitative research offered an opportunity to get a deeper understanding of the position of AI adoption in developing countries like Zimbabwe and the ethical issues surrounding its use. The ethical challenges noted in this study include data privacy, discrimination, bias, and unfavorable culturally insensitive applications. The study recommends the need for a holistic approach toward crafting AI frameworks and policies in higher education institutions. Such policies can help institutions become responsible through the fair, transparent, accountable, and ethical use of AI in higher education thus positioning libraries at the forefront of use of these ever-evolving technologies.

### Limitations of the study

The study was limited to selected experiences in Zimbabwean public university institutions. Findings from the private universities may differ and perhaps the preparedness and experiences of these institutions might be different. Secondly, the findings may be limited to a specific timeframe. There may be changes to the policies and frameworks of AI use in higher education thus completely changing the findings of this study.

## Data Availability

The raw data supporting the conclusions of this article will be made available by the authors, without undue reservation.
